# Complex Case of Aggressive Intra-abdominal Desmoid-type Fibromatosis Status Post Cholecystectomy

**DOI:** 10.7759/cureus.7193

**Published:** 2020-03-06

**Authors:** Jigarkumar Rangunwala, Juliana Sitta, Varsha Prakash, Kshama Vyas, Manohar Roda

**Affiliations:** 1 Radiology, University of Mississippi Medical Center, Jackson, USA; 2 Radiology, University of Mississippi Medical Center, Jackson, USA; 3 Pathology, University of Mississippi Medical Center, Jackson, USA; 4 Family Medicine, University of Mississippi Medical Center, Jackson, USA

**Keywords:** desmoid tumor, desmoid-type fibromatosis, desmoid tumor status post cholecystectomy, unresectable desmoid tumor, locally advanced desmoid tumor, aggressive desmoid tumor, intra-abdominal fibromatosis

## Abstract

Desmoid-type fibromatosis (DF), also known as desmoid tumor, is an extremely rare, benign, mesenchymal fibrous tumor with no potential for metastasis. It can arise from any part of the body, most commonly extra-abdominally. Intra-abdominal DF can present sporadically, in sites of previous trauma, surgical scars and irradiation, or in association with familial adenomatous polyposis and Gardner syndrome. Intra-abdominal DF is uncommon and especially rare after a common surgery like cholecystectomy. We report a rare case of a 67-year-old male who presented with a locally aggressive intra-abdominal DF in the gallbladder fossa, status post cholecystectomy. This progressively enlarging infiltrative enhancing solid mass in the gallbladder fossa on serial computed tomography and magnetic resonance imaging demonstrated gastric outlet obstruction, biliary obstruction, portal vein narrowing and encasement of hepatic artery. Diagnosis of DF in this postoperative setting was delayed and challenging due to uncharacteristic clinical presentation. Radiologists should be aware of this unusual diagnosis and spectrum of imaging findings to help in timely surgical management and planning.

## Introduction

Desmoid-type fibromatosis (DF) is a rare benign fibrous tumor that either presents as an indolent or locally aggressive mass. It is mostly encountered extra-abdominally, affecting the extremities, head, neck, chest and breast. Intra-abdominal DF can occasionally be seen at sites of prior trauma, scars and radiation therapy, or in association with familial adenomatous polyposis (FAP) and Gardner syndrome [1]. Locally aggressive DF can lead to complications like gastrointestinal obstruction and bowel ischemia [1,2]. We report a case of a 67-year-old male who presented with an infiltrative intra-abdominal DF in the gallbladder fossa status post cholecystectomy, associated with gastric outlet obstruction, biliary obstruction, portal vein narrowing and encasement of hepatic artery.

## Case presentation

A 67-year-old male complaining of persistent right upper quadrant (RUQ) pain for over 10 months, status post cholecystectomy. The pain started one month after cholecystectomy performed at an outside hospital. This pain was radiating to epigastric region and associated with nausea, vomiting and unintentional weight loss of about 30 lbs since the onset of the pain. At the time of his prior surgery, his gallbladder was found to be severely inflamed, with dense adhesions. Cholecystectomy was performed without any complications, and the patient was discharged safely.

**Figure 1:**
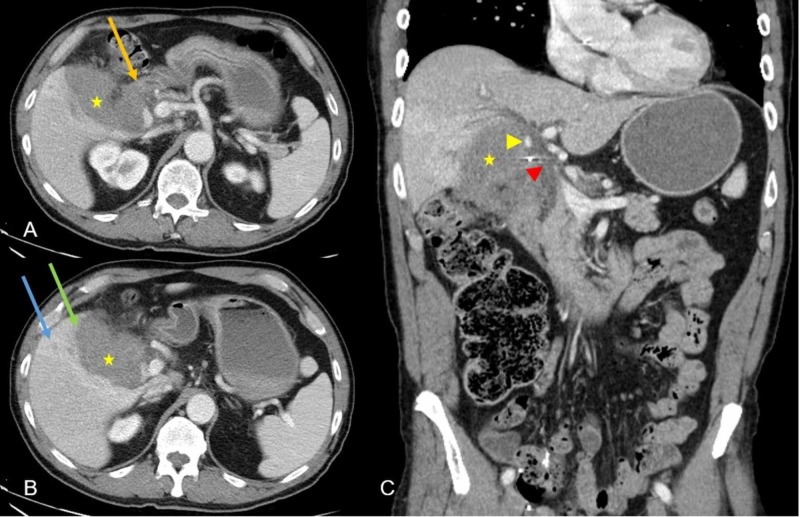
Mass in the gallbladder fossa. Multiple abdominal CT images: axial images (A, B) and coronal image (C) showing a lobulated, infiltrative, heterogeneously enhancing mass (yellow star) centered in the gallbladder fossa. Image (A) demonstrates loss of fat plane with proximal duodenum (yellow arrow). Image (B) demonstrates loss of fat plane with the inferior border of the right hepatic lobe (green arrow) and intrahepatic biliary dilation (blue arrow). Image (C) demonstrates compression and loss of fat plane with CBD (red arrowhead) and encasement of the right hepatic artery (yellow arrowhead). Hyperdense cholecystectomy clip is noted just above the red arrowhead. CBD, common bile duct

**Figure 2:**
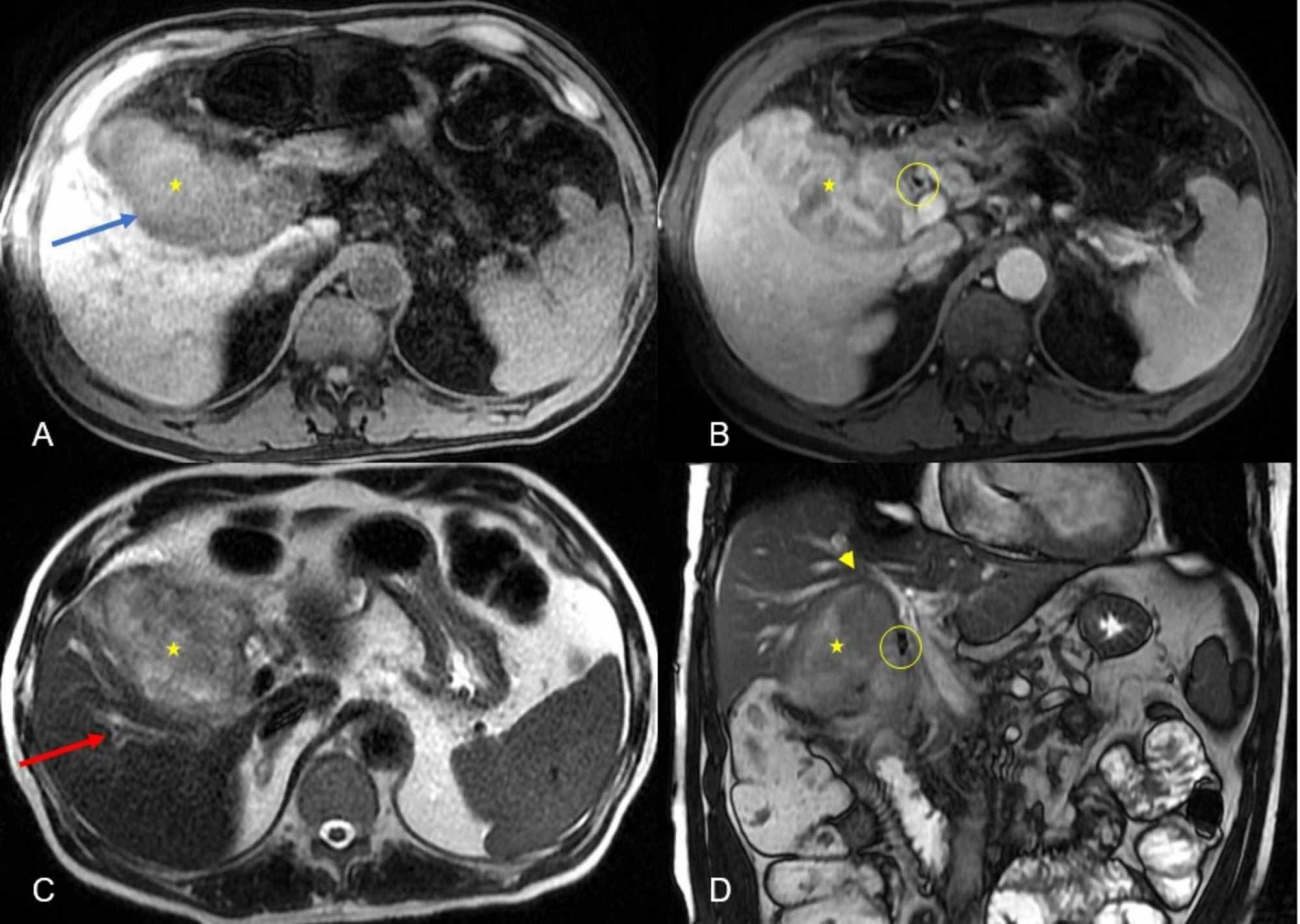
Infiltrative pattern and involvement of porta hepatis. Multiple abdominal MRI images: (A) axial T1 precontrast with fat suppression; (B) axial T1 postcontrast with fat suppression; (C) axial T2 and (D) coronal 2D FIESTA demonstrating a large lobulated mass in the gallbladder fossa (yellow star). On axial T1 image (A), the mass appears hypointense while on axial T2 image (C), it appears heterogeneously hypo/hyperintense (yellow star). The mass shows heterogeneous enhancement on postcontrast T1 image (B). There is infiltration of the inferior aspect of the right hepatic lobe (blue arrow on image A). The mass compresses the CBD with mild upstream right intrahepatic biliary dilation (red arrow on image C). There is compression and narrowing of the anterior branch of the right portal vein (yellow arrowhead on image D). Surgical clip (yellow circle on image D) from prior cholecystectomy. FIESTA, fast imaging employing steady-state acquisition; CBD, common bile duct

After the RUQ pain started, he underwent extensive workup at an outside hospital, which included computed tomography (CT), magnetic resonance imaging (MRI), magnetic resonance cholangiopancreatography, esophagogastroduodenoscopy and colonoscopy. Review of initial CT (Figure 1) and MRI (Figure 2), performed seven months after the onset of symptoms, revealed a heterogeneous enhancing solid mass in the gallbladder fossa. The mass exerted compression over porta hepatis structures, with associated encasement of the common bile duct (CBD) and right hepatic artery. There was also loss of fat planes with the right hepatic lobe and proximal duodenum.

Diagnostic laparoscopy and biopsy were performed at the outside hospital, which revealed predominantly fibrous soft tissue on pathology. At that time, it was concluded that this was a benign process not likely to result in postoperative scarring, and the patient was discharged with anti-inflammatory medications.

The patient subsequently presented at our hospital, with acute abdominal pain and symptoms of gastric outlet obstruction, leading to surgical consult. After review of his prior history and investigations, the patient underwent an exploratory laparotomy. At the time of surgery, the mass indeed appeared to cause gastric outlet obstruction and a gastrojejunostomy was performed. Complete excision was not possible due to unexpected increased size of this gallbladder fossa mass resulting in advanced infiltration to porta hepatis structures. Multiple excisional biopsies were taken for pathological analysis.

**Figure 3:**
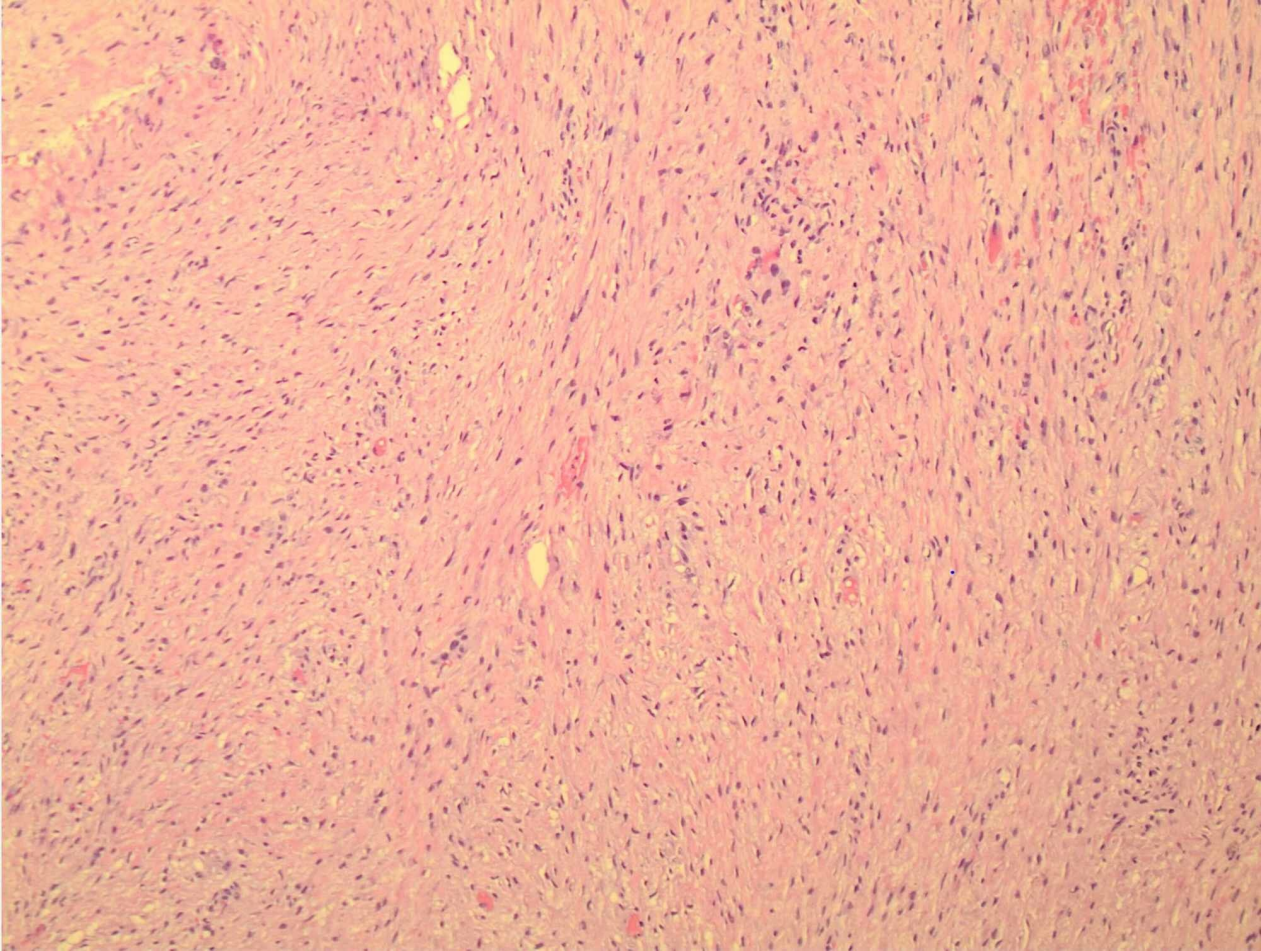
Hematoxylin and eosin stain of abdominal mass fragment. Core biopsy histology slide shows proliferative fascicles of spindle-shaped fibroblasts in an abundantly collagenized background. The fibroblasts are separated by thin-walled, delicate blood vessels.

**Figure 4:**
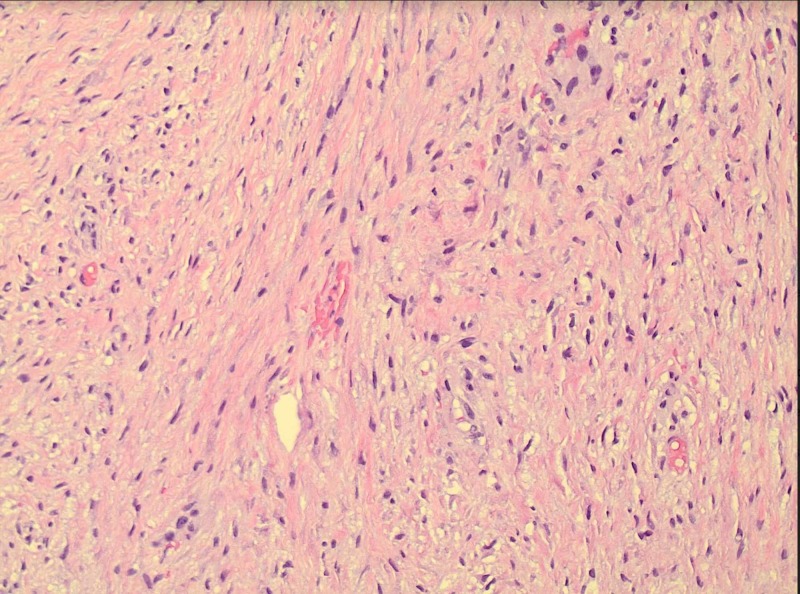
Spindle-shaped fibroblasts. The spindled fibroblasts exhibit low cellularity and bland cytology with no cellular atypia and mitotic figures. The nuclei are oval with fine chromatin.

**Figure 5:**
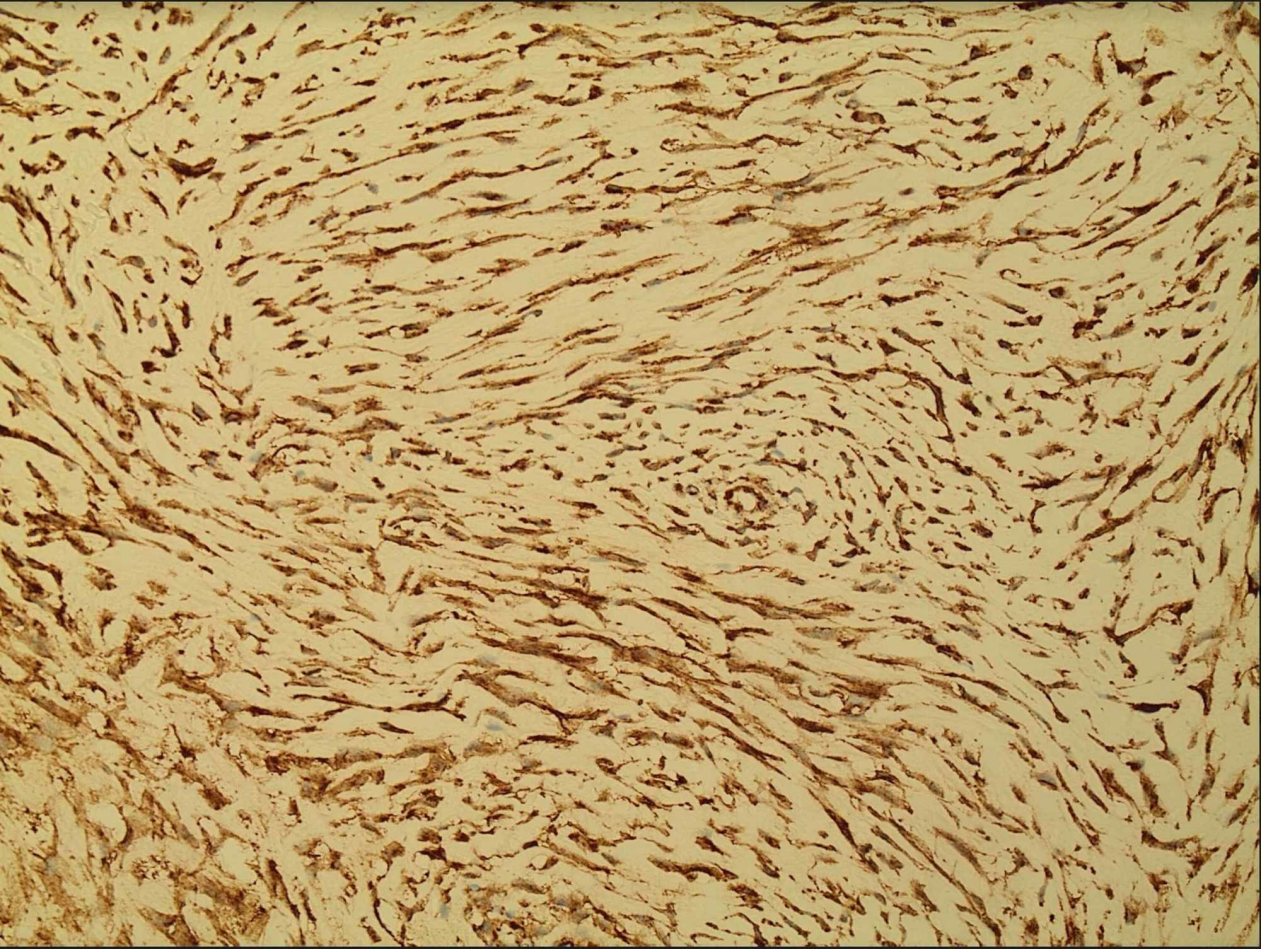
Beta-catenin immunohistochemistry. Positive β-catenin imunohistochemistry with diffuse fibroblast nuclear staining, faint cytoplasmic staining which is characteristic of desmoid fibromatosis.

The abdominal mass biopsies were stained with hematoxylin and eosin stain for histological examination. This revealed proliferative broad sweeping fascicles of spindle-shaped fibroblasts in an abundantly collagenized background. The fibroblasts were separated by thin-walled, delicate blood vessels that were aligned along edges of fascicles (Figure 3). The tumor cells were slender and relatively uniform, with a spindled configuration and abundant cytoplasm with indistinct cell borders. The nuclei were oval with fine chromatin and occasional small nucleoli. The spindled fibroblasts exhibited low cellularity and bland cytology with no cellular atypia and mitotic figures (Figure 4). On immunohistochemical analysis, the tumor cells were positive for β-catenin (Figure 5). Smooth muscle actin (SMA) was positively expressed in some regions. Overall, all these pathology findings are compatible with the diagnosis of desmoid fibromatosis.

**Figure 6:**
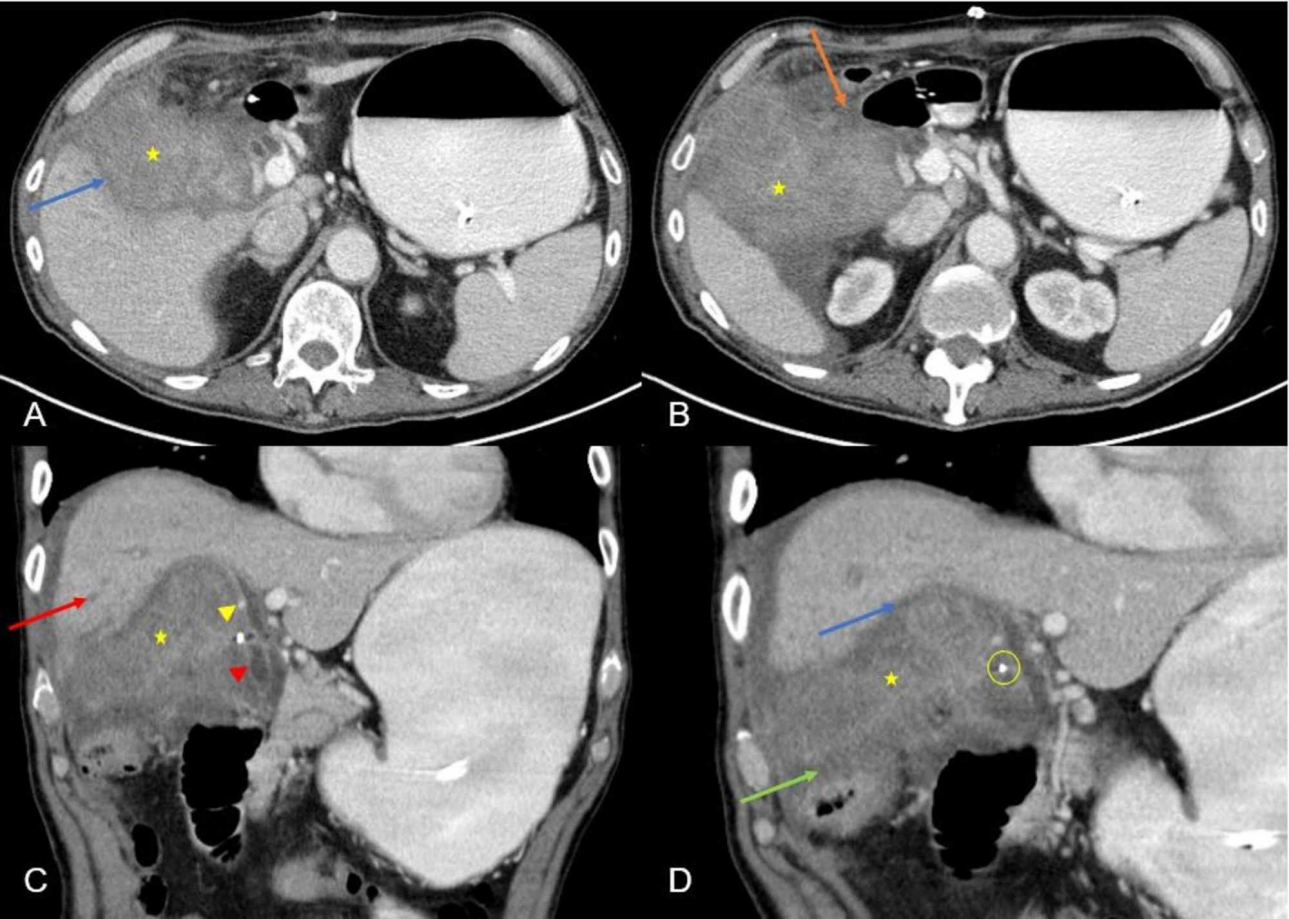
Interval increase in size of the mass. Multiple abdominal CT images: axial images (A, B) and coronal images (C and D) demonstrating interval increase in size of the mass with progressive infiltrative features in the gallbladder fossa (yellow star) resulting in involvement of multiple structures. There is loss of fat plane with the pylorus and proximal duodenum (orange arrow on image B) resulting in gastric dilation. There is loss of fat plane with the undersurface of the liver (blue arrows on image A and D). The mass is showing progressive compression of the CBD, right hepatic duct and inseparable from the cystic duct stump (red arrowhead on image C). There is upstream right intrahepatic biliary dilatation (red arrow on image C). There is encasement of the right hepatic artery at the porta hepatis (yellow arrowhead on image C) with compression of right portal vein. There is loss of fat plane with hepatic flexure (green arrow on image D). Surgical clip (yellow circle on image D) from prior cholecystectomy. CBD, common bile duct

Repeat post-operative CT performed shortly after the gastrojejunostomy demonstrated a significant interval increase in size of the mass (Figure 6), compared to outside CT performed three months ago. This mass now showed a significant increase in size and locally aggressive features with involvement of multiple structures: pylorus, proximal duodenum, undersurface of liver, right hepatic flexure, porta hepatis including CBD, right portal vein and right hepatic artery but with maintained patency. These findings resulted in gastric outlet obstruction, biliary obstruction and encasement of vascular structures.

The patient’s management and pathology was discussed at the hepatobiliary multidisciplinary conference in view of failed resection. The possibility of a complete tumor resection was excluded due to extensive local invasion and involvement of multiple structures and the porta hepatis. The decision was made in favor of nonsurgical management, imaging follow-up and trial of tamoxifen therapy as suggested by the oncologist.

## Discussion

DF, also known as aggressive fibromatosis, deep fibromatosis or desmoid tumor, is a rare sarcoma subtype that is benign and does not metastasize. It accounts for 0.03% of all neoplasms and 3% of all soft tissue neoplasms [2]. It is usually seen between 15 and 60 years of age with peak age of onset at 30 years. The pathogenesis of DF is not completely understood. Although most cases of intra-abdominal (mesenteric) fibromatosis are sporadic, but association with FAP and Gardner syndrome is suggestive of genetic predisposition. Trisomy of chromosomes 8 and 20 has been reported in several cases of DF, and trisomy 8 may be associated with an increased risk of recurrence [3]. These tumors are commonly associated with prior trauma, prior surgery, irradiation and high estrogen levels such as pregnancy [3,4].

The clinical presentation of DF is unpredictable, as it may range from being asymptomatic and indolent mass to locally aggressive mass with complications related to local obstruction and infiltration. It can arise from any mesenchymal tissue in the body, mostly seen extra-abdominally originating from musculoaponeurotic tissues [4]. CT and MRI are commonly used imaging modalities to assess and follow up DF. Initial ultrasound evaluation is usually reserved for selective superficial masses of the extremities [1]. On CT, intra-abdominal tumors are usually sharply marginated and extra-abdominal or mesenteric tumors may be seen with ill-defined infiltrative margins. Its variable attenuation is similar or slightly higher than skeletal muscle, likely reflecting the histological contents of different combination of collagen-myxoid elements. Postcontrast enhancement on cross-sectional imaging is commonly mild to moderate and calcifications are rare [1,5].

MRI is the preferred imaging modality to assess extra-abdominal and intra-abdominal DF. The MRI signal intensity pattern reflects the proportion of different tissues present microscopically. It most commonly presents heterogeneously with hypo/hyperintense signal on T2 images and iso/hypointense signal on T1 images. The band sign has been demonstrated on all MRI sequences for DF, described as hypointense nonenhancing linear bands, presumably representing dense collagen stroma. Although common, it is not a pathognomonic sign, as it can be seen in other soft-tissue tumors such as giant cell tumor of tendon sheath and myxofibrosarcoma. Enhancement after administration of gadolinium contrast media is commonly seen, particularly in hypercellular regions of the tumor. The value of diffusion-weighted MRI sequences is still unclear [1,5].

Although the imaging pattern will raise the possibility of DF, histological analysis is always required to rule out differential diagnosis. Immunohistochemical studies of desmoid tumors have demonstrated a fibroblastic-myofibroblastic phenotype. Muscle-specific actin, SMA and desmin are expressed in varying proportions of cases [6]. Coexpression of β-catenin and p53 may be associated with a higher risk of recurrence [7]. Morphologic variations of desmoid fibromatosis include hyalinized or hypocellular areas, dilated staghorn blood vessels, myxoid change, keloidal collagen bundles and hypercellular foci [8]. Dystrophic calcification is unusual. Mast cells can be scattered throughout the tumor but are more concentrated near blood vessels. A lymphocytic infiltrate may be seen peripherally at the interface between the desmoid and adjacent soft tissue. Atrophic or multinucleated skeletal muscle cells can simulate cellular atypia. Most desmoid tumors have relatively low mitotic rates, but occasional examples may display more than 10 mitoses per 10 high-power fields; this has no prognostic significance [9].

Surgery is the standard treatment for DF in symptomatic patients without significant morbidity. However, for high-risk patients and locally advanced infiltrative masses, complete excision could lead to a potential loss of function, and increased morbidity and mortality. Moreover, the literature is controversial regarding the prognostic significance of either positive or negative margins for DF tumors [10]. Progression free survival has been seen in up to 50% of patients in five years with conservative approach in several retrospective studies. Currently, a case-based conservative management approach has been recommended [11]. Many nonsurgical options have been used, such as hormone therapy, radiotherapy, cryoablation and chemotherapy. When critical structures are involved and hormonal therapy (tamoxifen) fails, a chemotherapy approach is prudent with either a low-dose regimen with methotrexate and/or vinblastine/vinorelbine or an anthracycline-based regimen [10,11].

## Conclusions

DF is a rare entity with peculiar imaging features, resulting in delayed diagnosis. Radiologists should be aware of this unusual diagnosis and spectrum of imaging findings including locally aggressive behavior. Early tissue sampling with laparoscopic/open biopsy is advisable for timely diagnosis, surgical management and ruling out other neoplasms.
